# MRPS16 facilitates tumor progression via the PI3K/AKT/Snail signaling axis

**DOI:** 10.7150/jca.39671

**Published:** 2020-02-03

**Authors:** Zhen Wang, Junjun Li, Xiaobing Long, Liwu Jiao, Minghui Zhou, Kang Wu

**Affiliations:** 1 Department of Neurosurgery, Tongji Hospital, Tongji Medical College, Huazhong University of Science and Technology, Jiefang Street, Wuhan 430030, P.R. China.; 2 Department of Neurosurgery, Union Hospital, Tongji Medical College, Huazhong University of Science and Technology, Jiefang Street, Wuhan 430022, P.R. China.; 3 Department of Neurosurgery, The First People Hospital of Qujing, Qujing 655000, P.R. China.

**Keywords:** glioma, invasion, MRPS16, PI3K/AKT/Snail

## Abstract

**Background**: Although aberrant expression of MRPS16 (mitochondrial ribosomal protein S16) contributes to biological dysfunction, especially mitochondrial translation defects, the status of MRPS16 and its correlation with prognosis in tumors, especially glioma, which is a common, morbid and frequently lethal malignancy, are still controversial.

**Methods**: Herein, we used high-throughput sequencing to identify the target molecule MRPS16. Subsequently, we detected MRPS16 protein and mRNA expression levels in normal brain tissue (NBT) and different grades of glioma tissue. The molecular effects of MRPS16 in glioma cells were tested by Western blotting, quantitative polymerase chain reaction (qRT-PCR), EdU, CCK-8, colony formation, Transwell migration and invasion assays.

**Results**: Intriguingly, we found that MRPS16 knockdown suppressed tumor cell growth, migration and invasion. Conversely, MRPS16 over-expression increased tumor cell growth, migration and invasion. In addition, subsequent mechanistic studies indicated that MRPS16 promoted glioma cell growth, migration and invasion by the activating PI3K/AKT/Snail axis. Furthermore, we observed that the decrease in tumor cell growth, migration, invasion and Snail expression mediated by MRPS16 knockdown could be rescued by Snail over-expression.

**Conclusion**: In short, our data demonstrate that MRPS16 over-expression remarkably promotes tumor cell growth, migration and invasion via the PI3K/AKT/Snail axis, which may be a promising prognostic marker for glioma.

## Introduction

Malignant glioma is the most frequent and lethal cancer derived from the CNS (central nervous system), accounting for approximately 42% of intracranial tumors, and characterized by rapid development and poor prognosis 1-3. Although the diagnostic methods and treatment strategies for glioma have improved undeniably, the five-year survival rate is not very optimistic. Tumor progression is the main cause of this phenomenon 4,5. Therefore, better understanding of the specific molecular mechanism that underlies tumor progression is crucial to improving glioma diagnosis and treatment.

Recently, a growing number of publications have focused on the biological function of MRPS16. MRPS16 encodes a 137-amino acid protein that is the most conservative ribosomal proteins from mammalian to yeast mitochondria. The human protein shares approximately 40% sequence identity with its bacterial homologs 6. The human mitochondrial ribosome contains two subfamilies: the small subfamily (SSU) and the large subfamily 7. The binding of ribosomal protein S16 is a vital step in the SSU assembly 8, which occurs on a narrow crevice of the SSU. The extraribosomal function of introducing nicks into supercoiled DNA molecules is necessary 9. For the presence of a premature stop codon located in MRPS16 is expected to lead to a truncated and likely unstable protein. The harmful effects of the mutation are evident by the sharp decrease in 12S rRNA levels. A missense mutation in the mitochondrial ribosomal protein 12S in Drosophila, which could interfere with the protein assembly in the active ribosome, led to an obvious decrease in 12S rRNA levels 10. All of these reports further indicate that MRPS16 plays vital roles in biological processes. However, its roles in cell proliferation, invasion and migration, the key features of glioma, remain elusive. Hence, based on findings of this research, we report that MRPS16 promoted glioma progression via the PI3K/AKT/Snail axis.

## Materials and methods

### Cell lines and reagents

Normal human astrocyte (NHA) cells, human astrocyte (HA) cells, and U-87MG (glioblastoma of unknown origin), U-138MG and U-251MG cells were cultured as stated in our previous reports 11,12. BEZ235 (2-methyl-2-(4-(3-methyl-2-oxo-8-(quinolin-3-yl)-2,3-dihydroimidazo[4,5-c]quinolin-1-yl)phenyl)propanenitrile) and LY294002 (2-morpholino-8-phenyl-4H-chromen-4-one) were purchased from Sigma-Aldrich. Anti-MRPS16 antibody (ab151693) and anti-Snail antibody (ab216347) were purchased from Abcam. For more information about all other antibodies, see our previous article 12.

### Patients

A total of 61 surgically removed tumor tissues and 4 cases of epilepsy brain tissue were collected at Tongji Hospital from September 2015 to June 2018 after receiving the informed consent of patients and the approval of the local Ethics Committee (TJ-IRB20181104). None of the patients had received radiotherapy or chemotherapy before surgery. All specimens were collected and stored in strict accordance with relevant regulations.

### Plasmid construction and transfection

The MRPS16 cDNA (GenBank Accession Number: NM_016065.4) and the Snail cDNA (NM_005985.4) were cloned into pcDNA3.0 to construct the over-expression plasmid pcDNA3.0-MRPS16 and pcDNA3.0-Snail. The empty vector was used as a negative control. The plasmids were constructed by Shanghai GeneChem Co., Ltd, China. The MRPS16 target sequence was 5'-CACCTCTCTAAGCCTATGGAA -3' and the Snail target sequence was 5'-GCTGAGCTGTTACTAGGACAA-3'. For transfection assays, U-138MG and U-87MG cells were transfected with corresponding plasmids. Detailed procedures were described in our previous article 11. Briefly, U-138MG and U-87MG cells were routinely cultured in six-well dishes to 85% confluence. All the cells were transfected with MRPS16-sgRNA or Snail -sgRNA or empty vector according to the manufacturer's protocols. The MOI (multiplicity of infection) value of this experiment was 10. After 48 h of transfection, the stable cell lines were selected by treatment with 1 µg/ml puromycin for 2 weeks.

Three short hairpin RNAs were designed based on the MRPS16 sequence. We picked the one with the biggest knockout effect. The short hairpin RNA 5'-GCTGAGAGACTGCCAAGGAAA-3' was designed by Gene (Shanghai, China) based on the MRPS16 sequence. The oligoduplexes were cloned into the pLKO.1-TRC cloning vector and then transfected into U-138MG and U-87MG. Similar to the above, transfected cells were selected with 1 µg/ml puromycin for 2 weeks.

### Cell migration and invasion experiments

Cell migration and invasion experiments were performed with the Transwell system (Corning, NY) following the manufacturer's instructions. Detailed procedures were described in our previous articles 11,12. To evaluate the invasion ability, filters were coated with Matrigel (BD Biosciences, USA). Approximately 6×10^5^ cells in serum-free DMEM were added to the top chamber, and the bottom chamber was filled with DMEM containing 10% FBS. The same experimental design was adopted for migration assays except that the filters were not precoated with Matrigel.

### CCK-8 assay

For the Cell Counting Kit-8 (CCK-8) experiment, cells were cultured in 96-well plates (5×10^3^/well) with complete culture medium. After treatment with CCK-8 (10μl), the absorption rate was determined using a microplate reader. More information on the detailed procedure in included in our previous article 11.

### Colony formation assay

Five hundred cells were resuspended and cultured in complete culture medium for two weeks, and the culture medium was not changed. Detailed procedures were described in our previous article 11,12. At least three independent experiments were performed.

### Western blotting

Proteins were separated by electrophoresis and then transferred to a PVDF membrane, which was blocked using 2% fetal bovine serum. Next, the membrane was incubated with the corresponding antibodies, and then antigen-antibody complexes were probed using an Enhanced Chemiluminescence Plus kit (Thermo). More information on the detailed procedure is included in our previous article 11.

### Co-IP (Coimmunoprecipitation)

Infected U-138MG and U-87MG cells were collected and resuspended in IP buffer. Then, the cell lysates were centrifuged (12000 g/20 min) and incubated with the corresponding antibodies covalently linked to protein A/G-agarose beads (Beyotime Institute of Biotechnology, Jiangsu, China), with IgG antibody as a negative control. Before the addition of antibodies, a certain proportion of each supernatant (Input) was obtained for use as a positive control. The pellets were washed five times with IP buffer. The proteins were collected from beads by boiling in SDS-PAGE loading buffer (BOSTER, Wuhan) and then subjected to Western blot.

### qRT-PCR

Total RNAs were isolated from tissues collected previously. Synthesis of cDNA and RT-PCR was performed according to the manufacturer's instructions. The sequences of the MRPS16 primers were as follows: forward, 5'-ACTCGTTGCCCTCAACCTAGA-3'; reverse, 5'- GCAGTCTCTCAGCATTTGTGA -3'. The sequences of the Snail primers were as follows: forward, 5'- ACTGCAACAAGGAATACCTCAG- 3'; reverse, 5'- GCACTGGTACTTCTTGACATCTG -3'. The relative mRNA expression level was standardized as described earlier 12-14.

### Cignal finder cancer 10-pathway reporter array

All operations were as reported in the previous literature 15. Briefly, the resuspended cells (5×10^5^/ ml, 50 μl/well) were plated into 96-well plates and were transfected with the luciferase reporters targeting common cancer pathways. Then, the cells were incubated, and the luciferase activity was detected.

### EdU proliferation assay

Newly synthesized DNA after the indicated treatments of U-138MG and U-87MG cells was measured by EdU fluorescence staining based on the manufacturer's instructions (Click-iT^®^ EdU Imaging Kits, Invitrogen). The cells, cultured in 96-well plates at a density of 1 × 10^5^ cells/well, were labeled with 10 μM EdU, incubated for 2.5 h, and then fixed for 15 min with 4% formaldehyde at room temperature. The fixative was removed, and the cells in each well were washed three times with 3% BSA in PBS. The BSA was removed, and the cells were permeabilized with 0.5% Triton X-100 (Sigma, San Francisco, CA, USA) for 25 min at room temperature. After washing the cells three times with 3% BSA in PBS, a 100 μL 1 × Click-iT^®^ reaction cocktail was added into each well, and the plate was incubated for 30 min at room temperature in the dark. Then, 1 ml of 1× Hoechst 33342 nuclear staining solution (Sigma, San Francisco, CA, USA) was added into each well, and the plate was incubated for 20 min at room temperature in the dark. Subsequently, the staining solution was removed, and the cells were washed three times with PBS. Then, the EdU-labeled cells were imaged and counted using a fluorescence microscope (CKX41-F32FL, Olympus, Tokyo, Japan). Image-Pro Plus software (version 5.0, MD, USA) was used to determine the percentage of EdU-positive (EdU+) cells.

### Brain orthotopic xenografts

Five mice (female nude mice, 15-17 g) were included in each group. The mouse head was fixed using a stereotactic apparatus, and the skull over the right hemisphere of the brain was exposed via a skin incision. The skull was drilled using a high-speed air-turbine drill (a burr tip size of 0.5 mm in diameter) until a bone flap became loose. Approximately 8 μL of U-138MG-Luc or U-87MG-Luc (MRPS16/Snail-luciferase, vector-luciferase, shMRPS16-luciferase or shControl-luciferase) cell suspension (8 × 10^6^ cells/ml in DMEM) that stably expressed firefly luciferase was injected into the brain parenchyma using a microliter syringe. Then, the bone flap was placed back and sealed with histocompatible cyanoacrylate glue. Subsequently, the scalp was sutured closed. Tumors were monitored and quantified using a bioluminescence imaging system (PerkinElmer, IVIS Spectrum Imaging System, USA) after four weeks. For bioluminescence imaging, mice received D-Luciferin Firefly, sodium salt (10 µl/g, 15 mg/ml, Glod Biotechnology, Inc., 10 min before imaging) and were anesthetized with 1% pentobarbital, followed by imaging with an IVIS spectrum imaging system. All animal experimental procedures were approved by the Institutional Animal Care and Use Committee of Tongji Medical College (S830).

### Immunohistochemical and immunofluorescence staining

All the procedures were as reported in the previous literature 11,12. In short, formalin-fixed and paraffin-embedded samples were deparaffinized, rehydrated, incubated and blocked with 10% normal goat serum. Then, the slides were incubated with the corresponding primary and secondary antibodies. Immunohistochemical scoring was performed according to the previous literature 12.

### Statistical analysis

Statistical significance was determined using Student's t tests. Statistical analysis was performed with GraphPad Prism 7. A two-tailed t-test or one-way analysis of variance (ANOVA) was used to identify statistical significance. The data are presented as the mean ± SD, with P<0.05 considered significant.

## Results

### MRPS16 over-expression is associated with poor prognosis in human glioma

**Figure 1:**
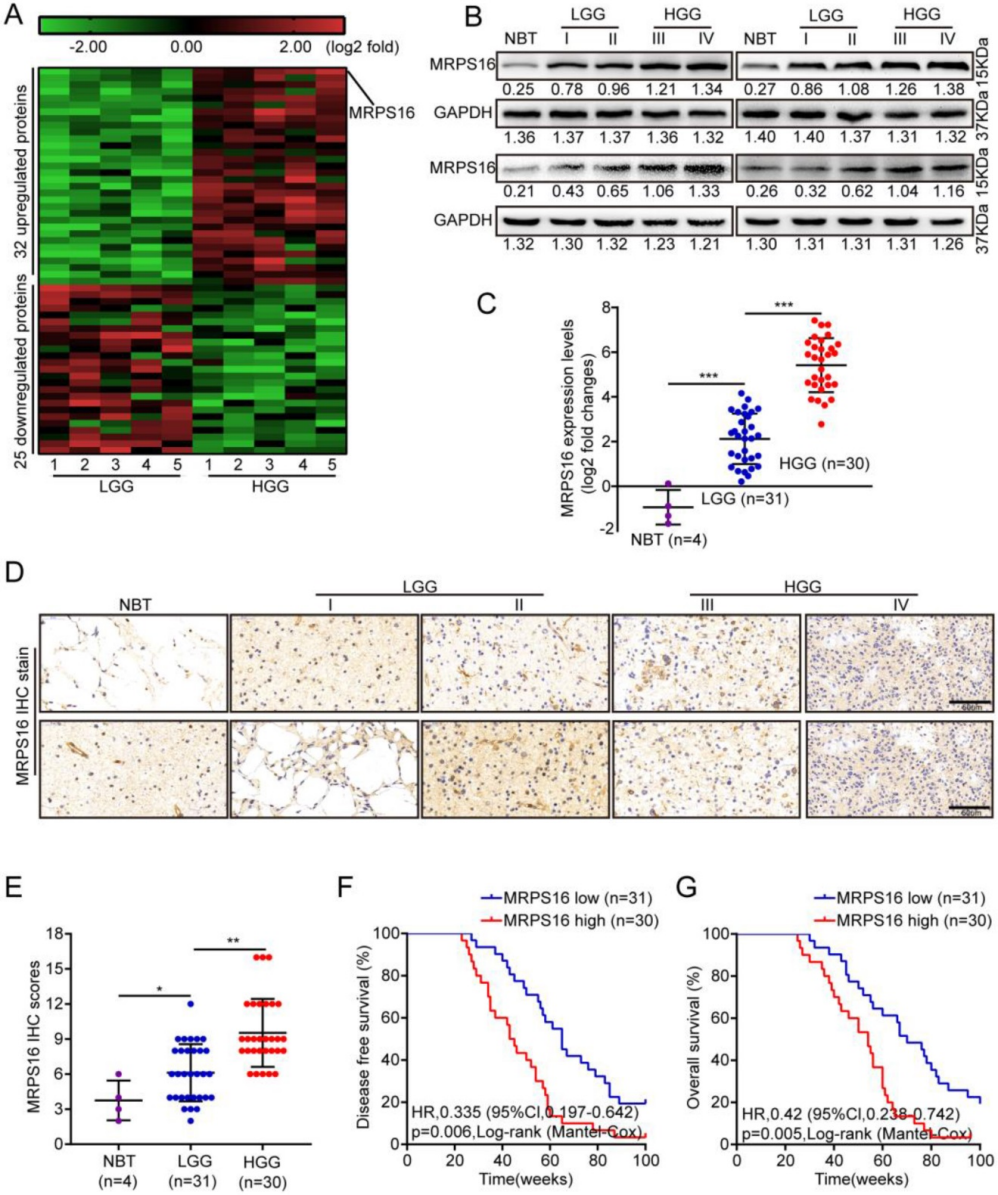
**MRPS16 over-expression is associated with poor prognosis in human glioma. A.** Unsupervised hierarchical clustering of the proteins differentially expressed in Low-grade glioma (LGG) and High-grade glioma (HGG). The pseudocolor represents the intensity scale of LGG vs. HGG, generated by a log2 transformation (fold changes > 5.0, p < 0.01). **B.** Western blotting for MRPS16 protein expression levels in frozen lysates from 4 normal brain tissue (NBT) and 16 different grades of glioma tissue. **C.** Relative MRPS16 mRNA expression levels were measured by RT-PCR in 4 normal brain tissue (NBT) and 61 different grades of glioma tissue. **D.** Representative immunohistochemical staining for MRPS16 protein expression levels in normal brain tissue (NBT) and different grades of glioma tissue. Bars: 50μm. **E.** MRPS16 protein expression levels scores are displayed as dot plots. **F - G.** Kaplan-Meier analysis showed a negative correlation between poorer disease-free survival or overall survival rates and MRPS16 mRNA expression levels in glioma patients. Statistical significance was tested using one-way ANOVA (Dunnett's tests) for multiple comparison and two-tailed t-tests. *P < 0.05, **P < 0.01 and ***P < 0.001.

To identify critical proteins that lead to glioma development, next-generation sequencing (NGS) was performed for five paired low-grade gliomas (LGGs) and high-grade gliomas (HGGs). As Fig. 1A shows, 32 and 25 proteins were up-regulated and down-regulated, respectively, by more than five-fold in HGG compared with LGG. First, MRPS16 protein and mRNA expression levels in normal brain tissue (NBT) and different grades of glioma tissue were measured by Western blotting and qRT-PCR. The data showed that MRPS16 often exhibited higher expression in tumor tissues, especially for HGG, compared with NBT (Fig. 1B and 1C). Then, we performed IHC to detect MRPS16 protein expression levels in NBT and different grades of glioma tissue. As Fig. 1D shows, the MRPS16 IHC intensity was significantly enhanced in tumor tissues, especially for HGG compared with NBT. Quantitative analysis further indicated that MRPS16 protein expression levels significantly increased in tumor tissues, especially for HGG compared with NBT (Fig. E). Then, we analyzed the correlation between MRPS16 mRNA expression levels and clinicopathological characteristics in 61 glioma samples. The data indicated that the MRPS16 mRNA expression levels were strongly related to the Karnofsky Performance Scale (KPS) score (P = 0.01) and recurrence (P = 0.001, Table 1), which is in accord with KPS as an independent predictor for survival 16. As Table 2 shows, high MRPS16 mRNA expression levels were associated with tumor grade and tumor recurrence by univariate and multivariate Cox regression analyses. These data indicate that the MRPS16 mRNA expression levels are negatively correlated with glioma prognosis. Furthermore, the Kaplan-Meier analysis indicated that MRPS16 mRNA expression levels were significantly associated with poorer disease-free survival (DFS) and overall survival (OS) in glioma patients (Fig. 1F and 1G).

### MRPS16 over-expression facilitates U-138MG cell progression

**Figure 2:**
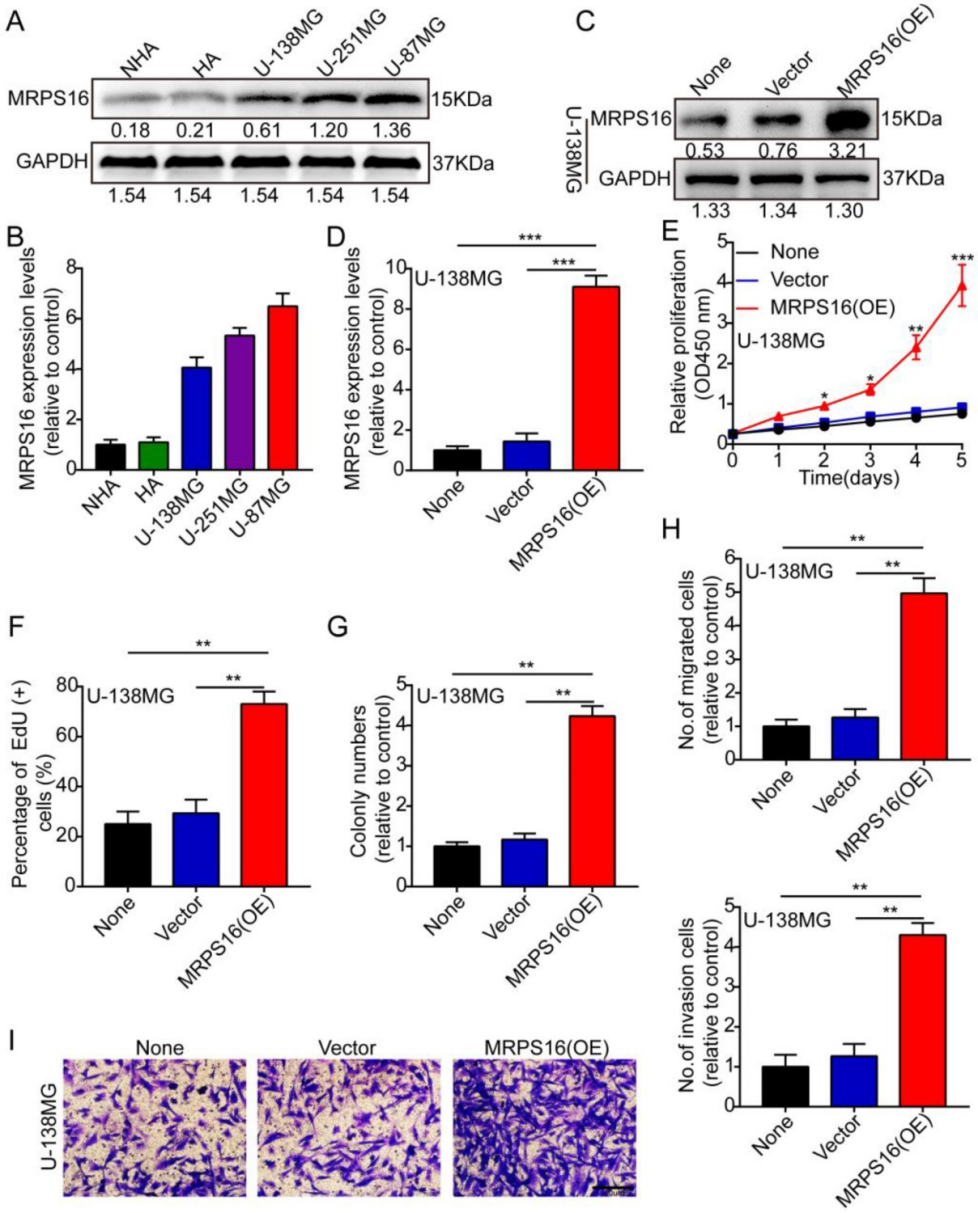
** MRPS16 over-expression facilitates U-138MG cells progression. A-B.** MRPS16 protein and mRNA expression levels by Western blot and qRT-PCR in NHA, HA, U-138MG, U-251MG and U-87MG. **C - D.** The over-expression efficiency for MRPS16 was verified in U-138MG by Western blot and qRT-PCR. **E.** Growth curves between None, Vector and MRPS16 (OE) by CCK-8 assay. **F**. MRPS16 over-expression promoted U-138MG cell proliferation. Percentage of EdU (+) is expressed in the panel. **G.** Over-expression of MRPS16 obviously facilitated colony formation and histogram quantification (panels). **H-I**. Transwell migration and invasion assay showing that over-expression of MRPS16 facilitated cell migration and invasion. The numbers of migrating and invading cells are measured in the panel. Bars: 50μm. Statistical significance was tested using one-way ANOVA (Dunnett's tests) for multiple comparison and two-tailed t-tests. All the results are shown as the Mean ± Standard Deviation (SD) of three independent repeated experiments at least. *P < 0.05, **P < 0.01 and ***P < 0.001. None: non infected cells.

First, we measured MRPS16 protein and mRNA expression levels by Western blot and qRT-PCR in two human brain gliocyte cell lines (NHA and HA) and three human brain glioma cell lines (U-138MG, U-251MG and U-87MG). The results demonstrated that MRPS16 protein and mRNA expression levels were up in the glioma cell line compared with the human brain gliocyte cell line (Fig. 2A and 2B). Subsequently, we over-expressed MRPS16 with lentiviruses targeting MRPS16 in U-138MG cells, and the over-expression levels of MRPS16 were measured by Western blot and qRT-PCR (Fig. 2C and 2D). Then, we adopted CCK-8, EdU, colony formation and Transwell experiments to determine the effects of MRPS16 on tumor cell growth, migration and invasion. The data showed that MRPS16 over-expression facilitated tumor cell growth, migration and invasion (Fig. 2E-2I and S1).

### MRPS16 knockdown suppresses U-87MG cell progression

**Figure 3:**
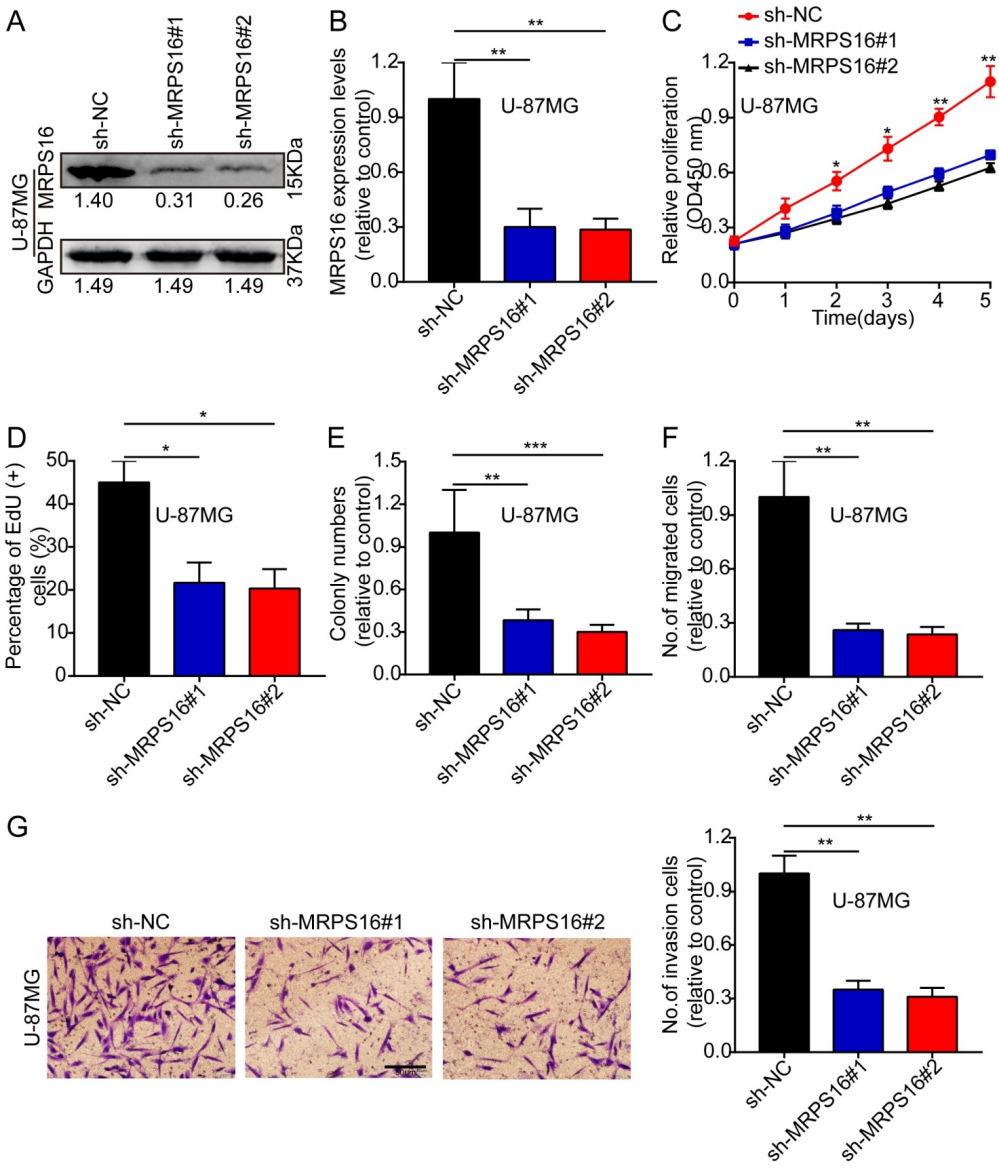
**MRPS16 knockdown suppresses U-87MG cells progression. A-B.** Validation of knocking down MRPS16 in U-87MG cells by Western blot and qRT-PCR. **C.** Growth curves between sh-NC, sh-MRPS16#1 and sh-MRPS16#2 groups by CCK-8 assay. **D.** Knockdown of MRPS16 suppressed U-87MG cell proliferation. Percentage of EdU (+) is expressed in the panel. **E.** Knockdown of MRPS16 suppressed colony formation and histogram quantification (panels).** F - G.** Transwell migration and invasion assay showing that knockdown of MRPS16 suppressed cell migration and invasion. The numbers of migrating and invading cells are measured in the panel. Bars: 50μm. Statistical significance was tested using one-way ANOVA (Dunnett's tests) for multiple comparison and two-tailed t-tests. All the results are shown as the Mean ± Standard Deviation (SD) of three independent repeated experiments at least. *P < 0.05, **P < 0.01 and ***P < 0.001. None: non infected cells.

Next, we knocked down MRPS16 with lentiviruses targeting MRPS16 in U-87MG, and the knockdown levels of MRPS16 were measured by Western blot and qRT-PCR (Fig. 3A and 3B). Then, we performed CCK-8, EdU, colony formation and Transwell experiments to determine the effects of MRPS16 on tumor cell growth, migration and invasion. These data showed that MRPS16 knockdown suppressed tumor cell growth, migration and invasion (Fig. 3C-3G and S2).

### MRPS16 promotes tumor progression via the PI3K/AKT/Snail axis

**Figure 4:**
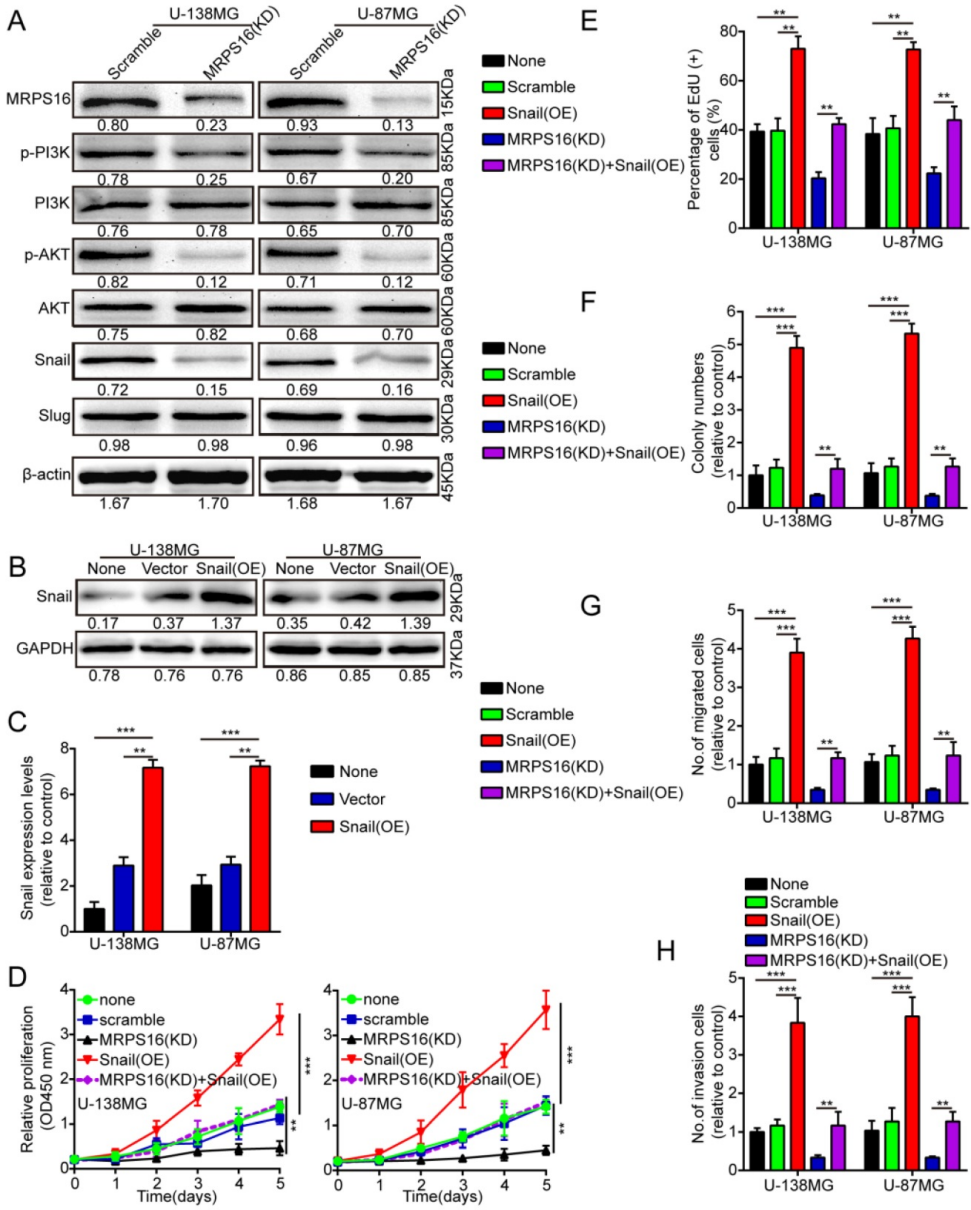
**MRPS16 promotes tumor progression by the PI3K/AKT/Snail axis. A.** Knockdown of MRPS16 decreased the protein levels of p-PI3K, p-AKT and Snail. **B**-**C.** Validation of over-expressing Snail in U-138MG and U-87MG cells by Western blot and qRT-PCR. **D.** Growth curves were tested by CCK-8 assay. **E.** Snail over-expression rescued the effects of MRPS16 knockdown in suppressing tumor cells growth. **F.** Over-expression of Snail rescued the effects of knockdown of MRPS16 in suppressing tumor cells colony formation. **G - H.** Transwell migration and invasion experiment was adopted as mentioned above. The numbers of migrating and invading cells are measured in the panel. Bars: 50μm. Statistical significance was tested using one-way ANOVA (Dunnett's tests) for multiple comparison and two-tailed t-tests. All the results are shown as the Mean ± Standard Deviation (SD) of three independent repeated experiments at least. **P < 0.01 and ***P < 0.001. None: non infected cells.

To clearly explain the underlying mechanism of MRPS16-mediated regulation of tumor progression, we performed the Cignal Finder Cancer 10-Pathway Reporter Kit to screen possibly involved signaling axes. Our results verified that PI3K/AKT signaling was notably inhibited, while other pathways were not obviously affected by MRPS16 knockdown in U-138MG and U-87MG cells (S3 E and F).We performed the dual luciferase reporter assay to verify the results. The reporter assay result was consistent with the previous observations, which indicated that MRPS16 could activate the PI3K/AKT signaling axis (S3 G and H). In addition, we also measured Snail and Slug protein expression levels, the downstream proteins of the PI3K/AKT signaling axis, using Western blot in U-138MG and U-87MG cells after MRPS16 knockdown. The Snail protein is involved in a series of cellular processes by a variety of different mechanisms 17,18. Snail over-expression can promote the migration, proliferation and invasion of squamous cell carcinomas 19. Therefore, we surmised that over-expression of MRPS16 might induce Snail expression, thus promoting glioma cell growth, migration and invasion. We found that the protein expression levels of Snail, p-AKT and p-PI3K significantly decreased after knockdown of MRPS16 in U-138MG and U-87MG cells (Fig. 4A). To verify whether Snail is involved in MRPS16-regulated glioma cell growth, migration and invasion, we over-expressed Snail in tumor cells (Fig. 4B and 4C). Snail over-expression rescued the effect of MRPS16 knockdown on the suppression of tumor cell proliferation (Fig. 4D-E and S3 A), colony formation (Fig. 4F and S3 B), migration (Fig. 4G and S3 C) and invasion (Fig. 4H and S3 D). These results showed that MRPS16 promotes tumor progression by the PI3K/AKT/Snail axis.

### MRPS16 binds to Snail and MRPS16 over-expression increases Snail via the PI3K/AKT pathway

**Figure 5:**
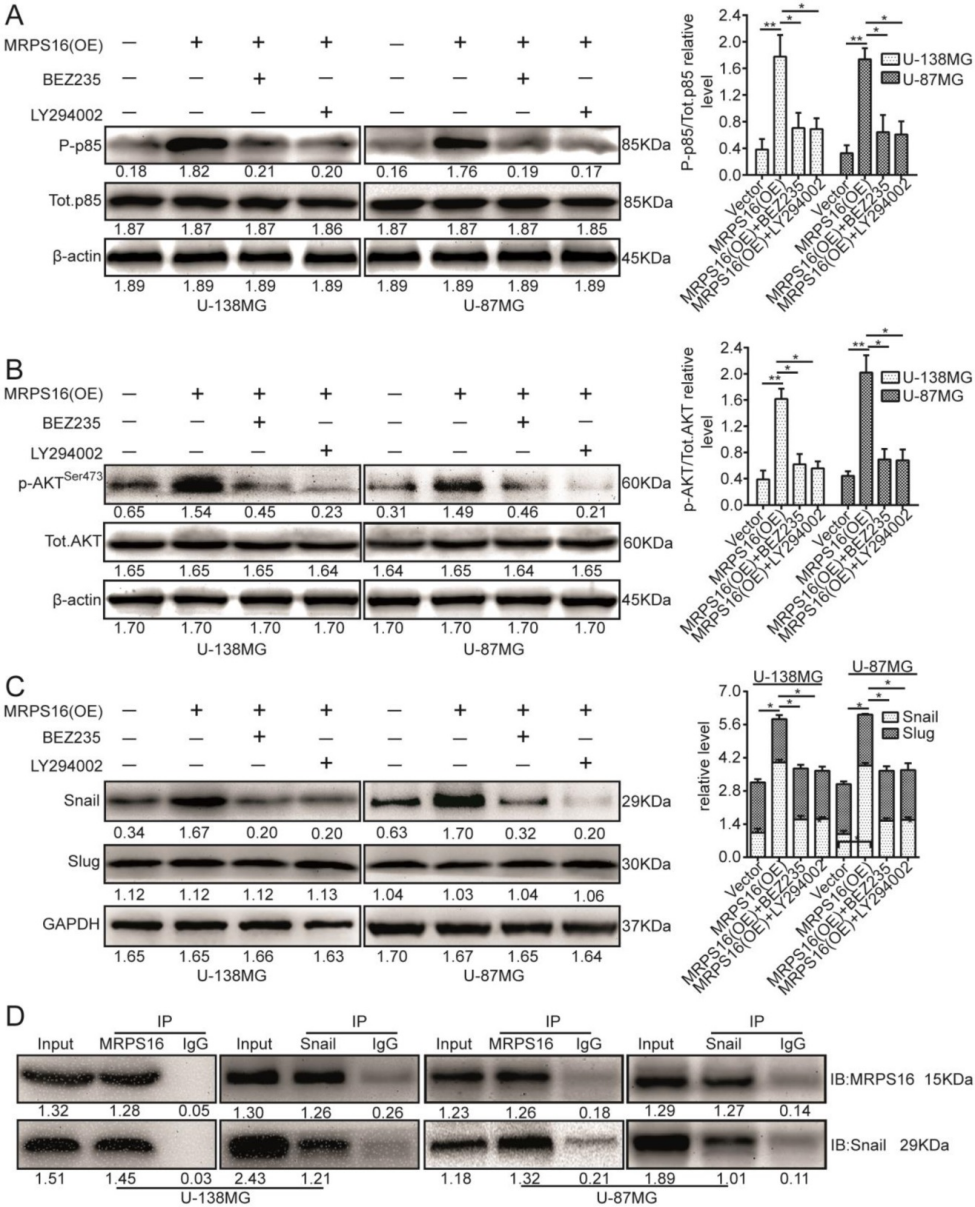
** MRPS16 over-expression increases Snail via PI3K/AKT pathway and binds with each other. A.** U-138MG and U-87MG cells transfected with MRPS16 plasmid and co-cultured with PI3K/AKT signaling inhibitors were reaped, and the lysates were immune-blotted for P-p85, Tot.p85 and β-actin. **B.** U-138MG and U-87MG cells transfected with MRPS16 plasmid and co-cultured with PI3K/AKT signaling inhibitors were reaped, and the lysates were immune-blotted for p-AKT^Ser473^, AKT and β-actin. **C.** U-138MG and U-87MG cells transfected with MRPS16 plasmid and co-cultured with PI3K/AKT signaling inhibitors were reaped, and the lysates were immune-blotted for Snail, Slug and GAPDH. **D.** Co-IP experiment indicated that MRPS16 could bind with Snail. Statistical significance was tested using one-way ANOVA (Dunnett's tests) for multiple comparison and two-tailed t-tests. * *P* < 0.05 and **P < 0.01.

The PI3K/AKT signaling axis is involved in a number of cellular processes by different mechanisms 20-22. However, it was unclear whether this signaling pathway is involved in the regulation of Snail by MRPS16. Therefore, we investigated whether MRPS16 over-expression promoted Snail protein levels via the PI3K/AKT axis. To verify this hypothesis, we cocultured MRPS16-overexpressing cells with two chemical inhibitors of the PI3K/AKT axis, BEZ235 and LY294002. First, their inhibition efficiencies in U-138MG and U-87MG cells were verified by p85 immunoblotting (Fig. 5A). The p-AKT^Ser473^ protein expression levels under MRPS16-overexpression in U-138MG and U-87MG cells were attenuated by the two inhibitors (Fig. 5B). Similarly, the Snail protein expression levels under MRPS16-overexpression were also reduced by the two inhibitors (Fig. 5C). Co-IP experiments indicated that MRPS16 could bind to Snail (Fig. 5D). All of these results convincingly showed that MRPS16-overexpression regulated Snail protein expression levels through PI3K/AKT axis.

### MRPS16 knockdown inhibits tumor growth

**Figure 6:**
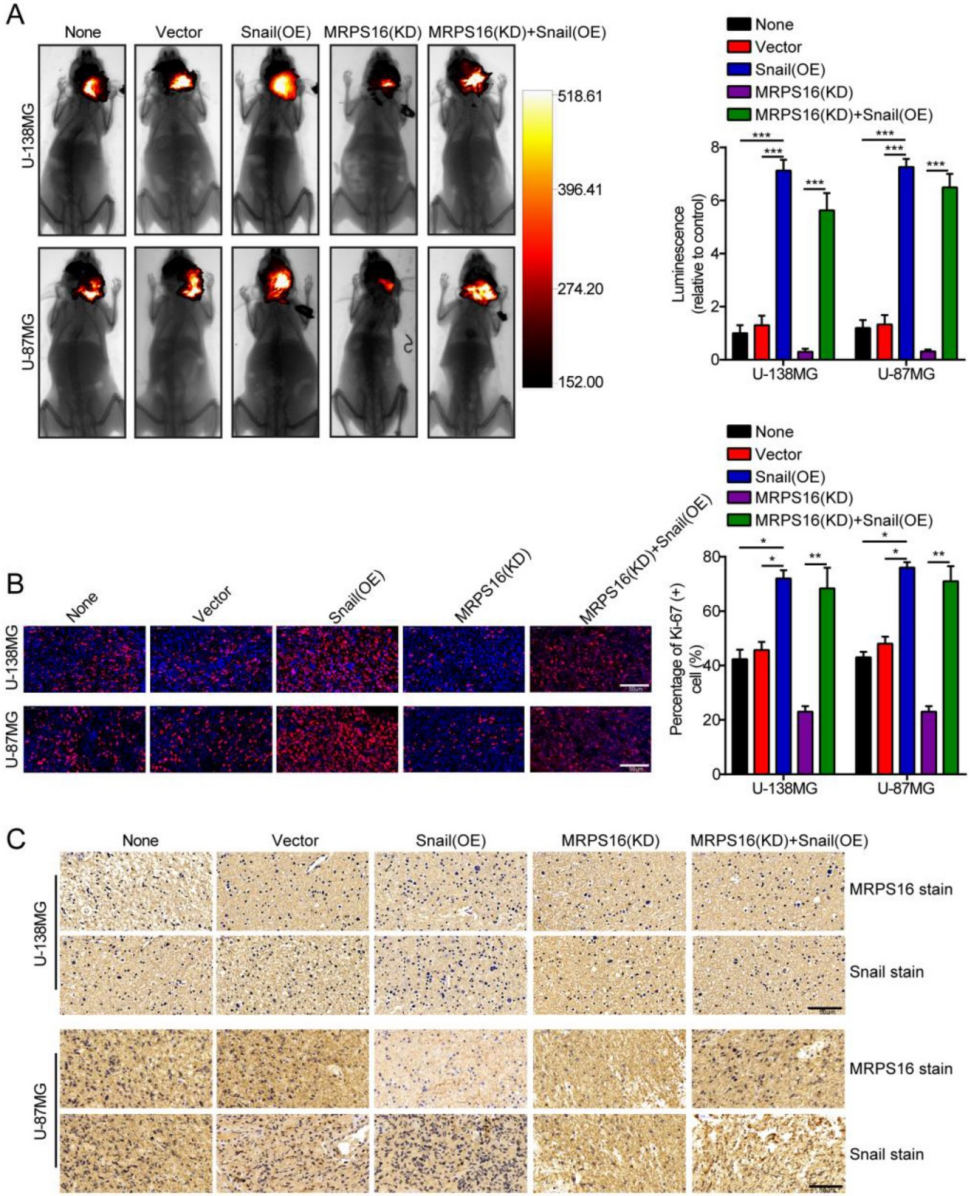
** MRPS16 knockdown inhibits tumor growth**. **A.** Representative images of the brain orthotopic tumor by U-138MG/Luc and U-87MG/Luc cells injected into the brain parenchyma of nude mice (left panels) and histogram analysis of luminescence representing the size of the tumors measured after four weeks (right panels). n = 5. **B**. Representative images (left panels) of IF staining with Ki-67 and histogram analysis of the percentage of Ki-67 (+) cells (right panels). **C**. Representative images of IHC staining with MRPS16 and Snail. Statistical significance was assessed using one-way ANOVA followed by Dunnett's tests for multiple comparisons. Scale bars: 50μm. *P < 0.05, **P < 0.01 and ***P < 0.001. None: non infected cells.

To further clarify the role of MRPS16 in glioma growth *in vivo*, a nude mouse orthotopic tumor model was established. The U-138MG/Luc and U-87MG/Luc cells stably over-expressing Snail or silenced for MRPS16 were inoculated into the brain parenchyma of nude mice (n = 5 per group). Four weeks after injection, the size of the tumors in the nude mice was quantified by measuring the luminescence intensity using a bioluminescence imaging system. The results indicated that MRPS16 knockdown could inhibit tumor growth and that Snail over-expression could abrogate the MRPS16 knockdown-mediated inhibition of tumor growth (Fig. 6A). IF staining indicated that MRPS16 knockdown suppressed the expression of Ki-67 compared with the control group. Conversely, Snail over-expression abrogated the MRPS16 knockdown-mediated inhibition of Ki-67 (Fig. 6B). In addition, IHC staining showed the protein expression levels of MRPS16 and Snail in each group (Fig. 6C). Collectively, all the data suggested that MRPS16 knockdown could inhibit tumor growth and that Snail over-expression could abrogate the effect *in vivo*.

## Discussion

Here, we report the clinical relevance and function of MRPS16 in glioma. We found that MRPS16 was often up-regulated in glioma tissues, and MRPS16 knockdown obviously suppressed tumor cell growth. Conversely, MRPS16 over-expression increased tumor cell proliferation. Hence, we proved that MRPS16 contributes to cancer development. Currently, accumulating evidence has focused on the function of MRPS16 proteins in mitochondrial translation defects; however, their function in glioma remains unknown.

Malignant gliomas are the most common and deadly brain tumors with a dismal prognosis 23-25. Although we have achieved certain results in the understanding of glioma over the years, the prognosis of glioma patients has not improved much. Therefore, it is very urgent to identify novel and efficient molecular markers to diagnose and treat patients with this disease.

Tumor progression is a multistep process involving many complicated mechanisms 26-28. Herein, we found the oncogenicity of MRPS16 in the development of glioma. According to our study, we verified that over-expression of MRPS16 promoted tumor cell growth, migration and invasion, which indicates that MRPS16 has strong tumorigenicity in glioma. However, the specific molecular mechanisms of MRPS16 underlying invasion remain unknown. Therefore, our data suggest that MRPS16 promotes tumor progression by modulating the protein expression of Snail. The high expression levels of Snail were related to tumor grade, metastasis and poor prognosis in patients with various cancers 29,30. In glioma cells, previous literature has reported that Snail can promote the progression of glioma 31,32. Interestingly, our previous research also proved that Snail can promote the progression of glioma 12. Our current research data also support previous literature reports. In addition, these data further clarified that MRPS16 facilitated tumor cell growth, migration and invasion by activating the PI3K/AKT/Snail axis. Previous literature also reported that activation of the PI3K/AKT signaling pathway promotes the progression of liver cancer by increasing the expression levels of Snail protein 33. Our experimental data also indicated that MRPS16 activated the PI3K/AKT signaling pathway, which in turn promotes the expression levels of Snail protein, thus promoting the progression of glioma. These results indicate that MRPS16 regulates Snail by activating the PI3K/AKT axis. According to our data and previous reports, we determined a model for how MRPS16 promotes glioma progression.

In conclusion, we propose and clarify that MRPS16 promotes glioma progression through the PI3K/AKT/Snail signaling axis and that these molecules may be potential new targets for the treatment of glioma.

## Supplementary Material

Supplementary figures.

## Figures and Tables

**Table 1:** Association of MRPS16 expression with clinicopathological characteristics in human glioma.

Features	No.	MRPS16		P-value
		Low	High	
**Age(years)**				
<50	33	17	16	0.91
>=50	28	14	14	
**Gender**				
Male	27	14	13	0.89
Female	34	17	17	
**Tumor size, cm**				
<3	37	24	13	0.006
>=3	24	7	17	
**Tumor location**				
Supratentorial	39	18	21	0.33
Subtentorial	22	13	9	
**Karnofsky performance scale**				
<90	38*	15	23*	0.01
>=90	22	16	6	
**WHO grade**				
Low-grade(I+II)	35	23	12	0.007
High-grade(III+IV)	26	8	18	
**Tumor recurrence**				
No	42*	27*	15	0.001
Yes	18	3	15	

* Partial data not available; statistics based on available data.

**Table 2:** Univariate and Multivariate analyses of various prognostic parameters in patients with glioma Cox-regression analysis.

	Univariate analysis				Multivariate analysis		
	p value	Hazard Ratio	95% confidence interval		p value	Hazard Ratio	95% confidence interval
**MRPS16**	0.023	1.469	1.252-2.342		0.02	1.535	1.392-2.778
**Tumor size, cm**	0.006	2.265	1.189-3.421		0.032	1.332	1.265-2.998
**Karnofsky performance scale**	0.01	1.767	1.436-2.849		0.012	1.612	1.253-1.899
**WHO grade**	0.007	2.135	1.376-3.132		0.042	1.211	1.147-2.476
**Tumor recurrence**	0.001	2.463	1.462-2.879		0.028	1.515	1.325-3.437
