# MRPS16 facilitates tumor progression via the PI3K/AKT/Snail signaling axis: Erratum

**DOI:** 10.7150/jca.104449

**Published:** 2025-01-01

**Authors:** Zhen Wang, Junjun Li, Xiaobing Long, Liwu Jiao, Minghui Zhou, Kang Wu

**Affiliations:** 1 Department of Neurosurgery, Tongji Hospital, Tongji Medical College, Huazhong University of Science and Technology, Jiefang Street, Wuhan 430030, P.R. China.; 2 Department of Neurosurgery, Union Hospital, Tongji Medical College, Huazhong University of Science and Technology, Jiefang Street, Wuhan 430022, P.R. China.; 3 Department of Neurosurgery, The First People Hospital of Qujing, Qujing 655000, P.R. China.

In the original version of our article, there were errors in Fig. 3, Fig. 6, Fig. S2 and Fig. S3. Specifically, the representative image of sh-MRPS16#1 cells in Figure 3G, the representative image of MRPS16(KD) groups in Fig. 6C, the representative image of sh-NC groups in Fig. S2C, and the representative image of Scramble groups in S3A are incorrect. The correct images are provided below. This correction will not affect the results and conclusions. The authors apologize for any inconvenience this may have caused.

## Figures and Tables

**Figure A:**
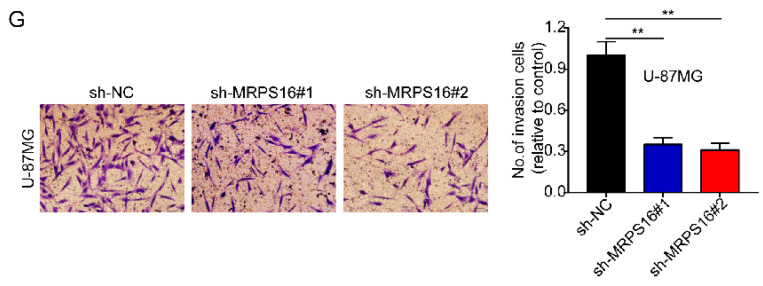
Correct image of Figure 3G.

**Figure B:**
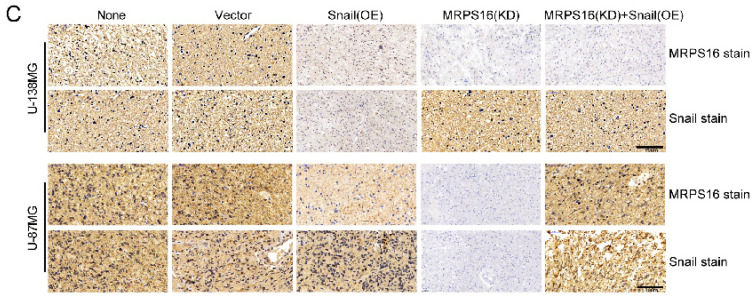
Correct image Figure 6C.

**Figure C:**
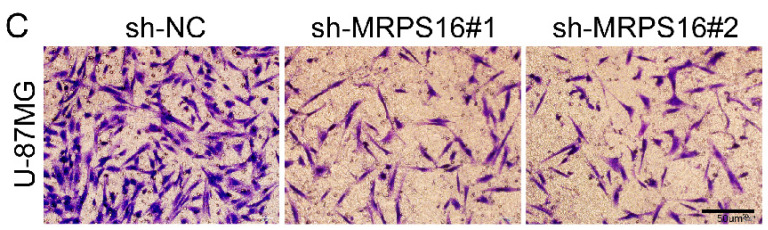
Correct image of Fig. S2C.

**Figure D:**
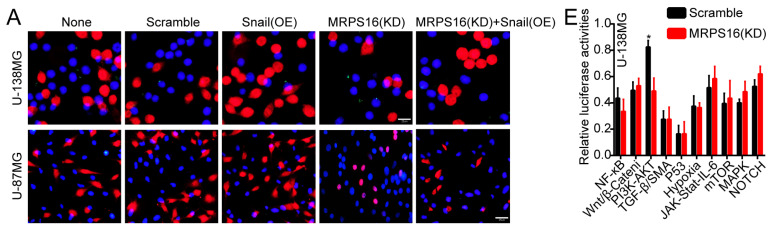
Correct image of Fig. S3A.

