# Supplementary material for: MRPS16 facilitates tumor progression via the PI3K/AKT/Snail signaling axis
Source: J Cancer. 2020 Feb 3;11(8):2032–43. doi: 10.7150/jca.39671 (PMC7052926; doi:10.7150/jca.39671)
Supplement: Supplementary file 1 — Supplementary figures. [file jcav11p2032s1.pdf]

MRPS16 facilitates tumor progression via the PI3K/AKT/Snail signaling axis

Zhen Wang[1#], Junjun Li[2#], Xiaobing Long[1], Liwu Jiao[3], Minghui Zhou[1], Kang Wu[1*]

S1. MRPS16 over-expression facilitates U-138MG cells progression.

MRPS16 over-expression promoted U-138MG cells proliferation, colony formation and migration. Representative images of the EdU (**A**), colony formation assay (**B**) and Transwell migration assay (**C**) with U-138MG cells.

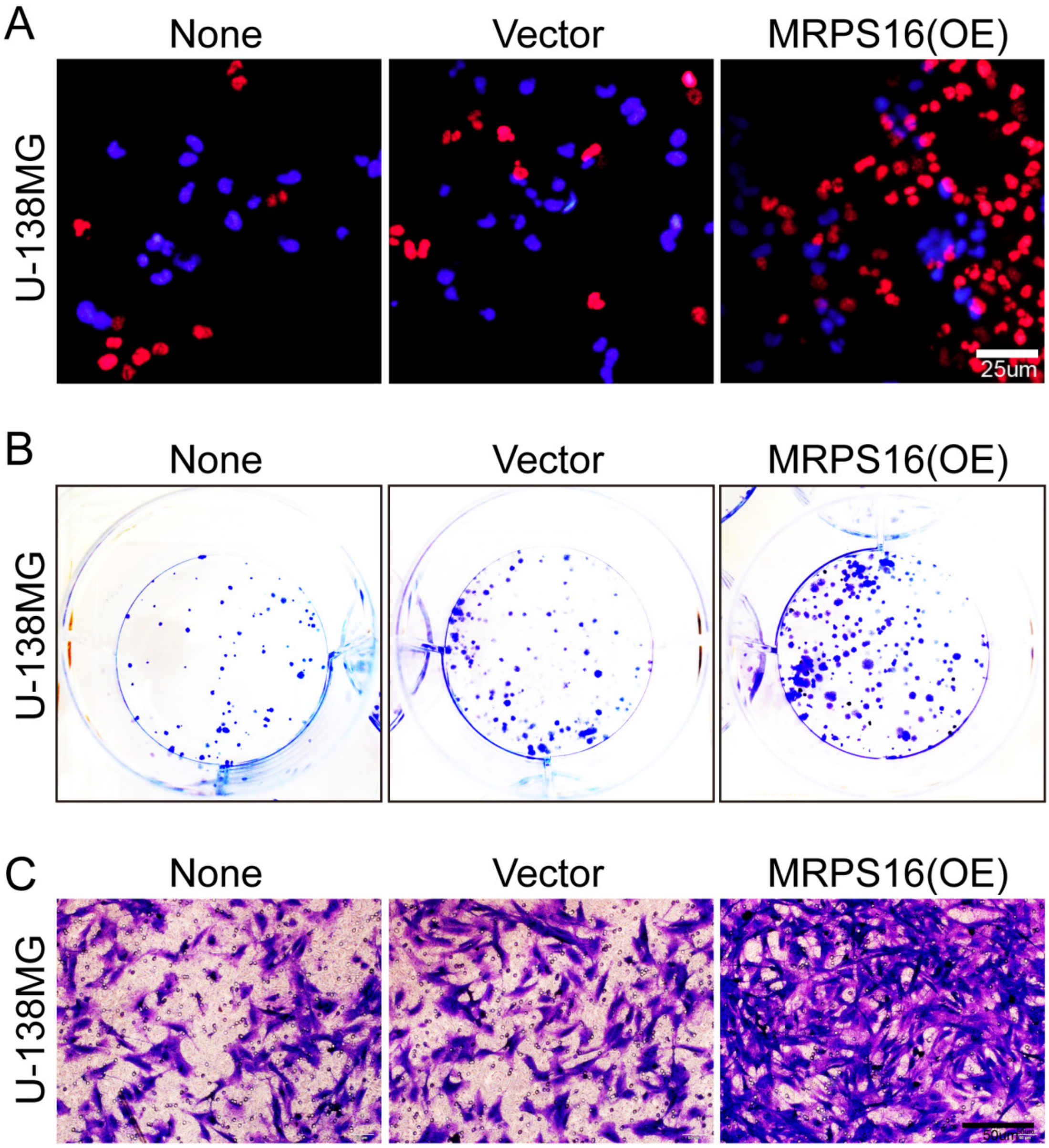

S2. MRPS16 knockdown suppresses U-87MG cells progression.

Knockdown of MRPS16 suppressed U-87MG cells proliferation, colony formation and migration. Representative images of the EdU (**A**), colony formation assay (**B**) and Transwell migration assay (**C**) with U-87MG cells.

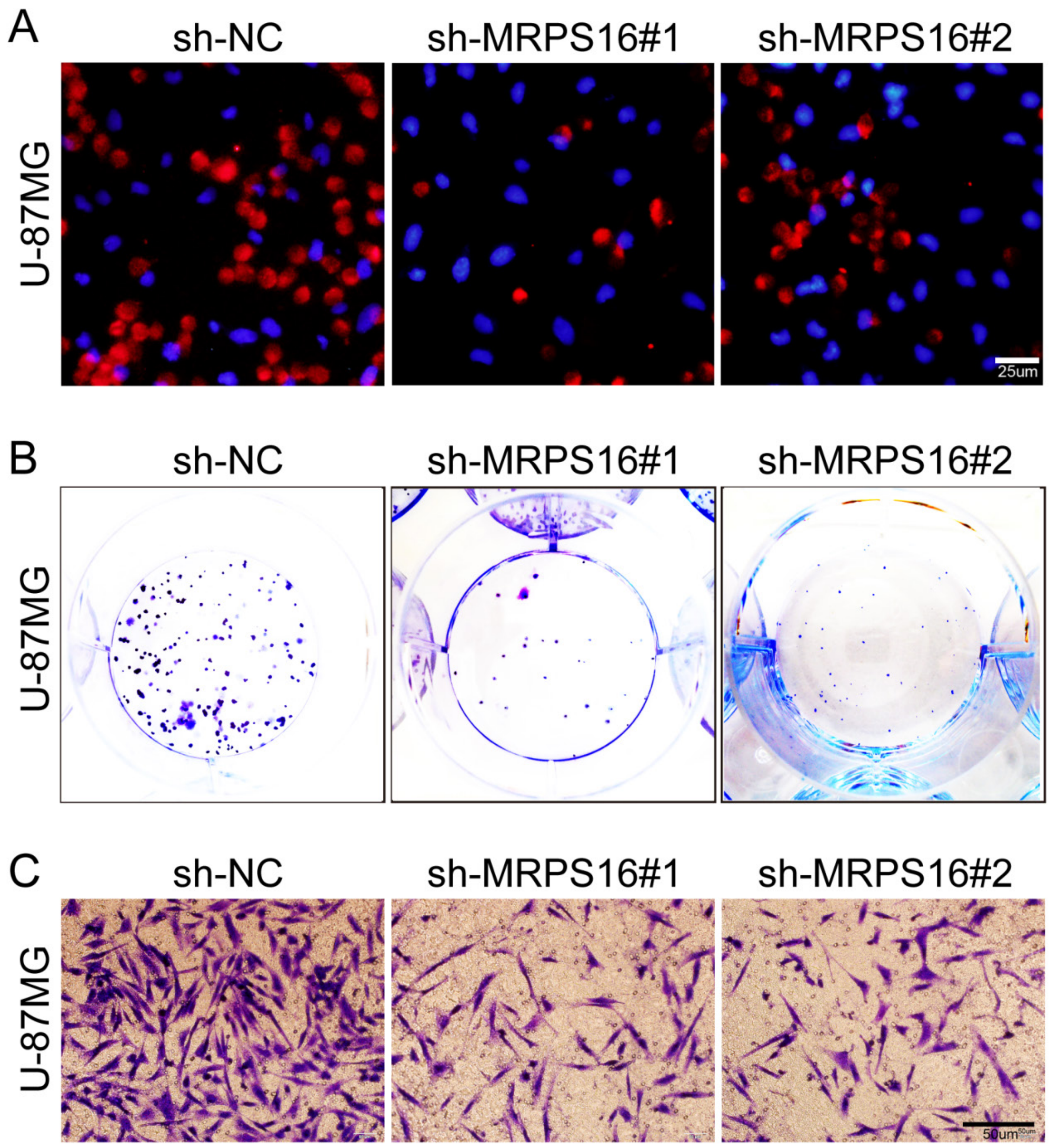

S3. MRPS16 promotes tumor progression by the PI3K/AKT/Snail axis.

Snail over-expression rescued the effects of MRPS16 knockdown in suppressing tumor cells growth, colony formation, migration and invasion. Representative images of the EdU (**A**), colony formation assay (**B**), Transwell migration (**C**) and invasion (**D**) assay with U-138MG and U-87MG cells. **E - F.** PI3K/AKT signaling axis was notably suppressed by MRPS16 knockdown, while others remained unchanged. **G - H.** The luciferase reporter assay indicated that MRPS16 knockdown suppressed the transcription activity of PI3K/AKT signaling in U-138MG and U-87MG cells.

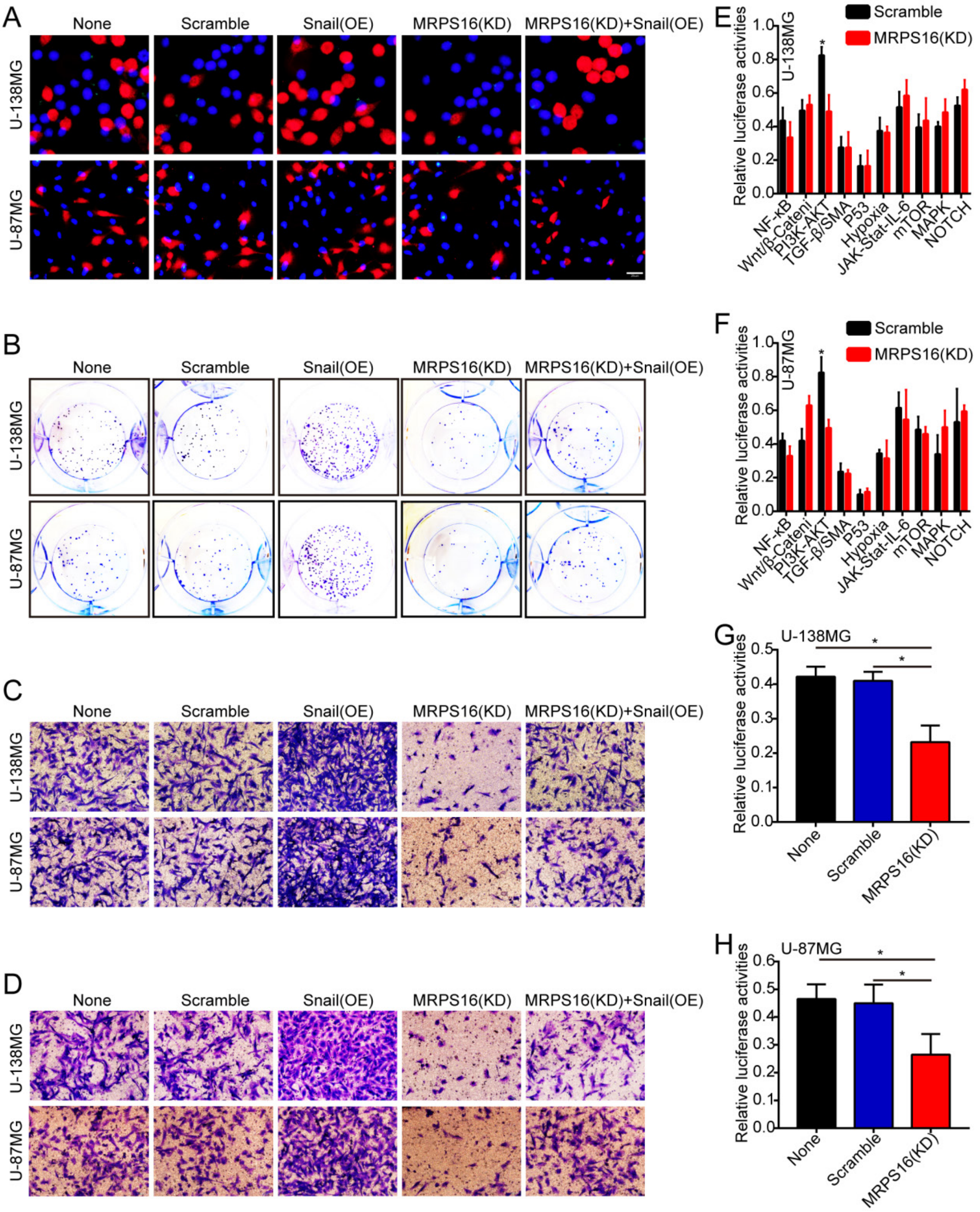

A
None
Scramble
Snail(OE)
MRPS16(KD)
MRPS16(KD)+Snail(OE)
U-138MG
U-87MG
B
C
D
E
F
G
H
Scramble
MRPS16(KD)
Relative luciferase activities
NF-κB
Wnt/β-Cateni
PI3K-AKT
TGF-β/SMA
P53
Hypoxia
JAK-Stat-IL-6
mTOR
MAPK
NOTCH
